# Surgery in Neonatal and Pediatric ECMO Patients Other Than Congenital Diaphragmatic Hernia Repair: A 10-Year Experience

**DOI:** 10.3389/fped.2021.660647

**Published:** 2021-05-04

**Authors:** Casper M. Kersten, Sergei M. Hermelijn, René M. H. Wijnen, Dick Tibboel, Robert J. M. Houmes, J. Marco Schnater

**Affiliations:** Department of Pediatric Surgery and Intensive Care, Erasmus University Medical Center Sophia Children's Hospital, Rotterdam, Netherlands

**Keywords:** extracorporeal membrane oxygenation, surgery, outcome, complications, pediatric, neonate, critical illness, post-surgical complications

## Abstract

**Aim of Study:** The use of extracorporeal membrane oxygenation (ECMO) has increased as a result of technological developments and the expansion of indications. Relatedly, the number of patients undergoing surgery during ECMO is also rising, at least in the adult population. Little is known on surgery in children during ECMO-therapy. We therefore aimed to assess the frequencies and types of surgical interventions in neonatal and pediatric patients on ECMO and to analyze surgery-related morbidity and mortality.

**Methods:** We retrospectively collected information of all patients on ECMO over a 10-year period in a single tertiary and designated ECMO-center, excluding patients undergoing cardiac surgery, and correction of congenital diaphragmatic hernia. Chi-squared test and Mann-Whitney U test were used to analyze data.

**Main Results:** Thirty-two of 221 patients (14%) required surgery when on ECMO. Common interventions were thoracotomy (32%), laparotomy (23%), fasciotomy (17%), and surgical revision of ECMO (15%). Complications occurred in 28 cases (88%), resulting in a 50% in-hospital mortality rate. Surgical patients had a longer ICU stay and longer total hospital stay compared to those not receiving surgery during ECMO. No significant difference in mortality was found when comparing surgical to non-surgical patients (50 vs. 41%).

**Conclusions:** Approximately one in seven neonatal or pediatric patients required surgical intervention during ECMO, of whom almost 90% developed a complication, resulting in a 50% mortality rate. These results should be taken into account in counseling.

## Introduction

Extracorporeal membrane oxygenation (ECMO) has been proven to be an efficient and cost-effective addition to conventional ventilator support in both children and adults (1–3). In the last decades, the use of ECMO has increased as a result of technical developments and an extension of indications, especially beyond the neonatal period. Relatedly, the number of patients undergoing surgery on ECMO is increasing. This increase is accompanied by higher complication rates in adults (4, 5). To date, complication rates and risks of surgical procedures in children on ECMO are still unknown, apart from those undergoing congenital diaphragmatic hernia (CDH) repair (6, 7).

Current indications for ECMO in the neonatal and pediatric populations include preoperative stabilization, post-surgical recovery, bridge to transplantation, bridge to recovery of organ function, and emergency cardiopulmonary resuscitation, so-called ECPR (8–10). Atkinson et al. (11) reported that 19 of 135 adults (14%) treated with ECMO underwent surgery while on ECMO in the years 1987 through 1989. Twenty-three years later, Taghavi et al. (5) reported a corresponding proportion of 269/563 (48%); an increase possibly demonstrating the effect of the extension of indications following new techniques and more experience. Surgical intervention during ECMO has not been associated with higher mortality rates in general (5, 11). Nevertheless, higher incidences of hemorrhage-related complications, due to the necessary anticoagulation during ECMO, have been reported (4, 5). The study of Taghavi et al. (5) found a significantly higher mortality rate in patients requiring blood transfusion because of hemorrhage.

In neonatal and pediatric patients, ECMO is often used as a bridge toward elective surgery of congenital abnormalities. Well-known examples are CDH, cardiac defects and bronchopulmonary abnormalities (4, 12–15). The role of ECMO in the management of CDH is debated and still subject of ongoing research (16). Although, clinical trials and subsequent systematic reviews have reported improved outcomes in CDH patients with the use of ECMO (2, 17, 18), repair of CDH on ECMO can lead to hemorrhage due to the necessary anti-coagulation (6, 7). Considering this, we decided not to include correction of CDH in this study.

To our knowledge, outcomes of neonatal, and pediatric patients undergoing surgical procedures on ECMO have not been published so far, apart from one abstract describing a cohort of 98 neonatal and pediatric patients on ECMO, 36 of whom (37%) underwent surgery. In-hospital mortality was not higher in the surgical group, but a longer median length of stay, a longer intensive care unit (ICU) stay and more blood transfusions were required following surgery (19). The exact complication rate of surgery on ECMO in neonatal and pediatric patients is unknown. Consequently, clinical decision making is mostly subjective and based on expert opinion rather than evidence. The aim of our study was to analyze the frequencies and types of surgical interventions in neonatal and pediatric patients on ECMO as well as the surgery-related morbidity and mortality.

## Methods

We searched our center's electronic patient database as well as the national Extracorporeal Life Support Organization (ELSO) database for patients who had received ECMO-treatment between January 2009 and January 2019 in our center. This University center is one of two neonatal and pediatric ECMO centers in the Netherlands, executing more than 30 ECMO runs each year. Data of all patients who had undergone one or more surgical intervention on ECMO—apart from insertion and removal of cannulas—were analyzed in detail. Patients undergoing CDH repair and cardiac surgery during ECMO represent a distinctly different population with electively planned surgery. This population was therefore found to be beyond the scope of this article and was excluded. In addition, patients undergoing surgery in another hospital were excluded due to missing data.

We collected the following information: the child's sex, gestational age at birth, and weight at start of ECMO, indication for ECMO, type of ECMO, and duration and number of ECMO-runs. Indication for ECMO was categorized into ECPR and respiratory or cardiac support, broken down for neonatal (<28 days age) and pediatric patients (≥28 days age), as is customary in the ELSO registry (10). Type of ECMO was categorized into veno-venous double lumen (VVDL), veno-arterial (VA), veno-venous (VV), a combination of types (hybrid), or multiple consecutive types (multiple).

Surgical procedures were categorized as thoracic, abdominal, and vascular. Only the surgical removal, replacement or placement of additional ECMO cannulas apart from primary installation of ECMO was counted as a surgical procedure. The number of days on ECMO elapsed at the time of the surgery was noted. Furthermore, we distinguished between therapeutic and diagnostic surgical procedures and elective vs. emergency surgery. The following outcome parameters were recorded: total length of hospital stay, total days at ICU and complications including mortality. Complications were categorized as hemorrhage, ischemia, compartment syndrome, mortality, and other. Cause of death was categorized as futility, neurological, cardiovascular, pulmonary, therapy failure, or directly related to surgery. Deaths were measured up until discharge from our center.

Perioperative anticoagulation was administered according to local protocol, which was updated in 2015. Preoperatively thrombocytes were required to be above 150 × 10^9^/L. Thirty minutes before start of the surgery Tranexamic acid was administered with a loading dosage of 4 mg/kg intravenously, followed by a continuous infusion of 1 mg/kg/h for 24 h or longer, depending on the extent of post-operative hemorrhage. Before 2015 Heparin was continued during surgery to maintain an activated partial thromboplastin time (APTT) of either 50–75 or 60–85 s depending on a normal or high thrombosis risk. After 2015 Heparin was stopped preoperatively, except when the risk of thrombosis was deemed high. Furthermore, fibrinogen levels were kept >1 g/L for 24 h after the surgical procedure (12, 20).

### Analysis

Statistical analysis was performed using SPSS (version 25, IBM Corp., Armonk, NY, USA). Differences in medians and percentages were assessed using the Mann-Whitney U test for continuous variables and the χ2 test for categorical variables.

## Results

**Figure 1:**
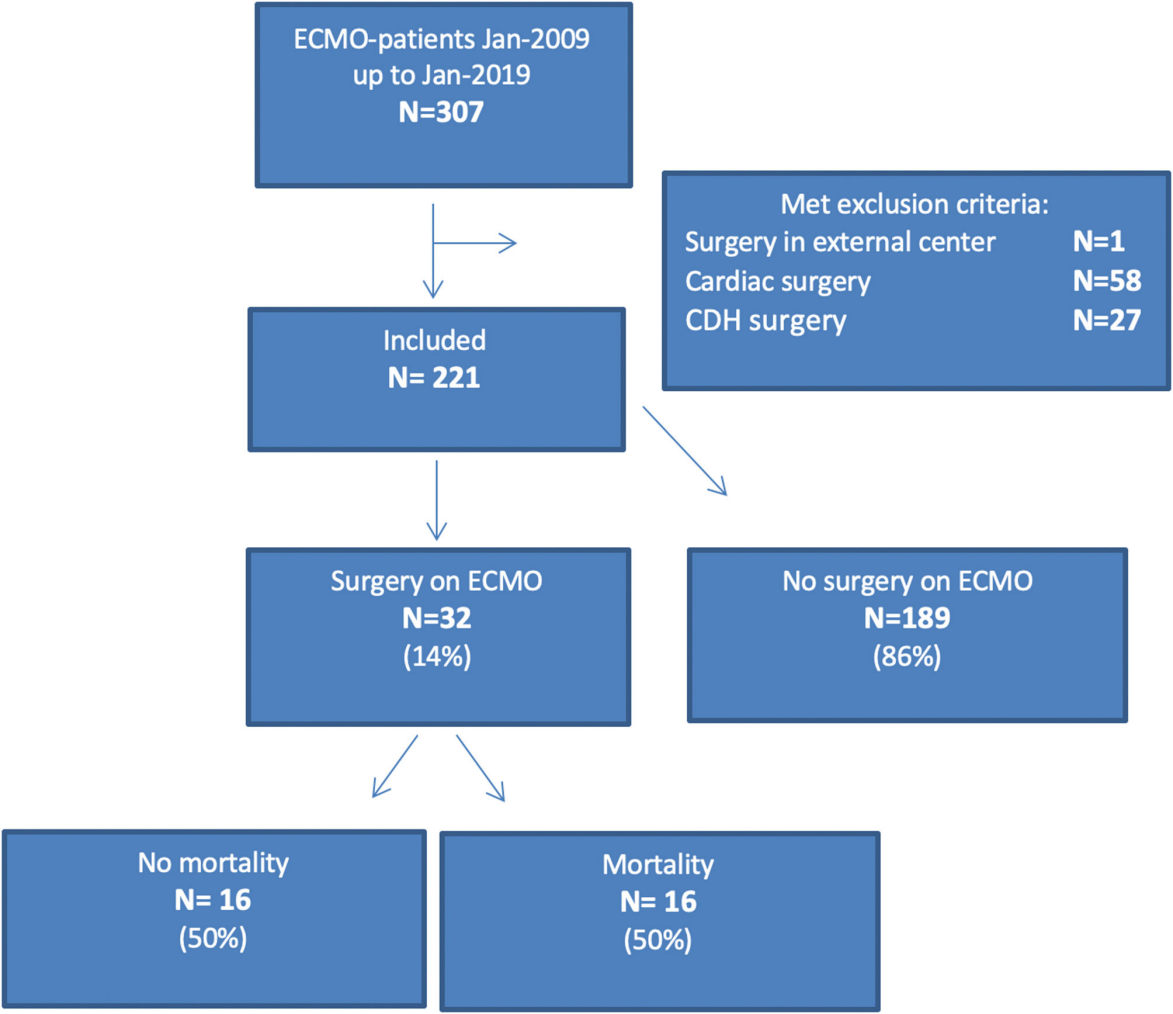
Flowchart.

In the 10-year study period, a total of 307 patients received ECMO-treatment in our center. Eighty-six patients were excluded; i.e., 58 (67%) who underwent cardiac surgery, 27 (31%) who underwent CDH repair, and one who underwent surgery elsewhere. Of the remaining 221 patients, 32 (14%) underwent surgery whilst on ECMO. See Figure 1 for the corresponding flowchart.

### Surgery vs. No Surgery (*n* = 221)

An overview of patient and ECMO characteristics is presented in Table 1. None of the baseline characteristics (sex, gestational age at birth, and birth weight) differed significantly between the surgical and non-surgical group.

**Table 1:** Baseline, ECMO, and outcome characteristics of surgical and non-surgical patients.

	**Surgery**	**No surgery**	***P*-value**
	***N* = 32 (14)**	***N* = 189 (86)**	
Male	18 (56)	106 (62)	0.986
Gestational age at birth	38.79 (31.71–41.71)	38.64 (25–42.43)	0.531
Birthweight	2700 (1800–4134)	3225 (500–5100)	0.356
Weight at start of ECMO^†^	14 (2.55–75)	4.2 (1.94–120)	0.005^*^
Age at start of ECMO categorical^†^			0.005^*^
premature <28 days	0	9 (5%)	
At term <28 days	7 (22%)	70 (37%)	
28 days−2 years	4 (13%)	47 (25%)	
>2 years	21 (66%)	63 (33%)	
ECMO indication^†^			0.144
Neonatal-respiratory	6 (19%)	61 (32%)	
Neonatal-cardiac	0	9 (5%)	
Neonatal-ECPR^‡^	1 (3%)	7 (4%)	
Pediatric-respiratory	13 (40%)	78 (41%)	
Pediatric-cardiac	3 (9%)	9 (5%)	
Pediatric-ECPR^‡^	9 (28%)	25 (13%)	
ECMO type^†^			0.127
VVDL	8 (25%)	80 (42%)	
VA	16 (50%)	85 (45%)	
VV	2 (6%)	9 (5%)	
Multiple	6 (19%)	15 (8%)	
ECMO duration^†^			0.006^*^
<7 days	12 (38%)	120 (64%)	
7–20 days	13 (41%)	55 (29%)	
>20 days	7 (22%)	14 (7%)	
ECMO run(s)^†^			0.707
1	30 (94%)	179 (95%)	
2	2 (6%)	6 (3%)	
3	0	3 (2%)	
4	0	1 (1%)	
ICU days^§^	22 (2–179)	12 (1–275)	0.003^*^
Total hospital days	24 (0–179)	15 (0–300)	0.023^*^
Complications			
Compartment syndrome	5 (16%)		
Other	3 (9%)		
Hemorrhage	2 (6%)		
Ischemia leg	2 (6%)		
Mortality on ECMO^†^	12 (38%)	53 (28%)	0.278
Mortality post-ECMO^†^	4 (13%)	24 (13%)	0.975
Complications total (including mortality)	28 (88%)		
Total in hospital mortality	16 (50%)	77 (41%)	0.327
Reason of death			0.69
Futility	8 (25%)	31 (16%)	
Neurological	2 (6%)	8 (4%)	
Cardiovascular	3 (9%)	19 (10%)	
Pulmonary	3 (9%)	13 (7%)	
Therapy failure	0	4 (2%)	
Surgical	0	1 (1%)	

*Data are reported as n (%) and median (minimum-maximum)*.

* *Indicates significance (p < 0.05)*.

† *Extracorporeal membrane oxygenation*.

‡ *Extracorporeal cardiopulmonary resuscitation*.

§ *Intensive care unit*.

#### ECMO Characteristics

Age and weight at start of ECMO were significantly higher in the surgical group compared to the non-surgical group. Furthermore, surgical patients had a significantly longer duration of ECMO compared to non-surgical patients.

#### Outcome

Both the length of stay on the ICU and the total length of hospital stay were significantly longer for the patients who underwent surgery on ECMO. However, the in-hospital mortality was not significantly different between the surgical and non-surgical group (41 vs. 50%, *p* = 0.327). While the mortality rate during ECMO was 38% in the surgical group compared to 28% in the non-surgical group, the mortality rate after cessation of ECMO was comparable between the two groups. Both differences were not statistically significant.

### Surgical Group

Thirty-two (14%) patients required surgery whilst on ECMO, 17 of whom (53%) received more than one intervention.

#### Types of Procedures

**Figure 2:**
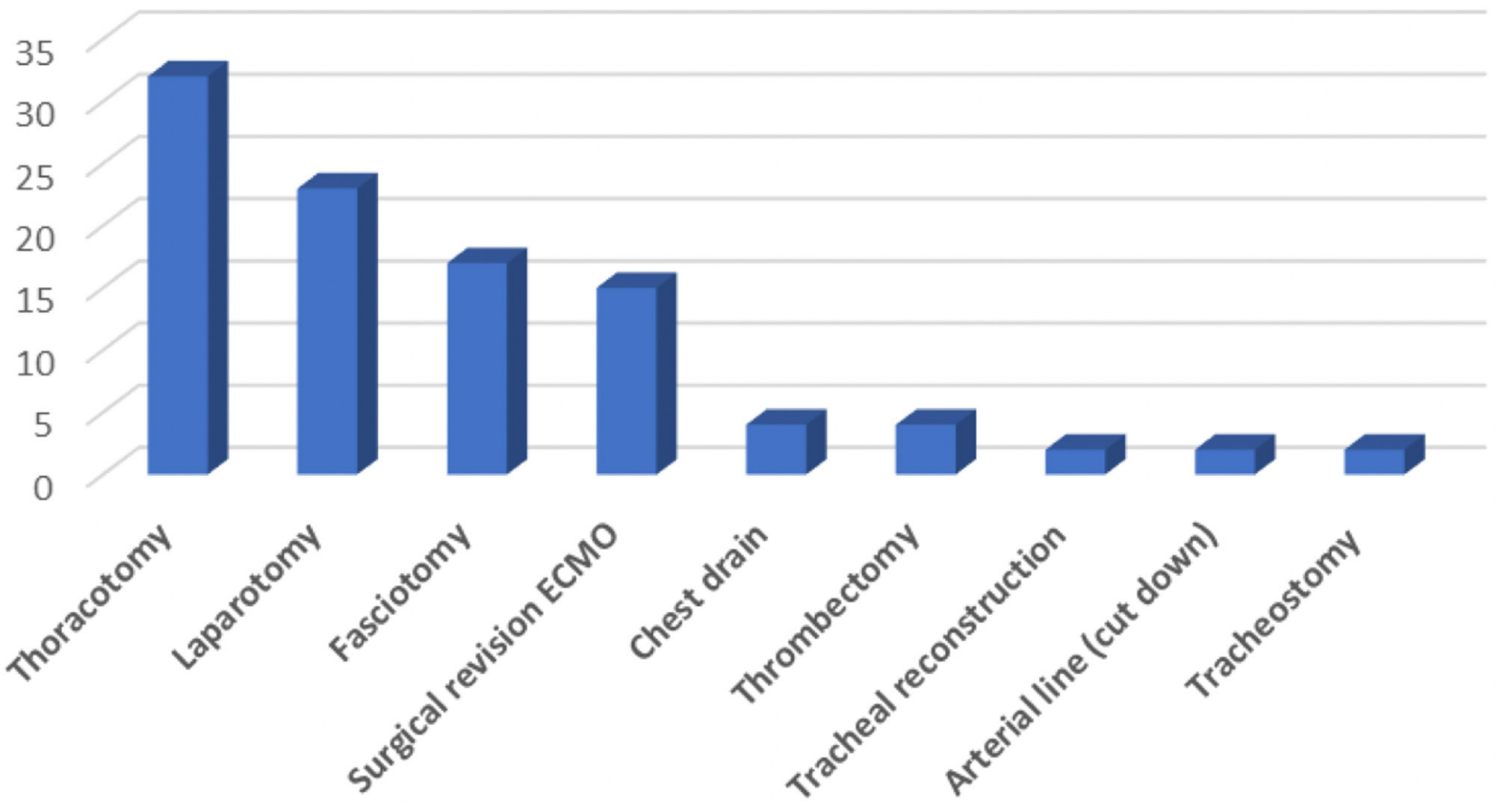
Surgical interventions during ECMO.

Thoracic surgery accounted for 40% of procedures, followed by vascular surgery (38%), and abdominal surgery (23%) (Table 2). In total 53 surgical procedures were performed in these 32 patients; thoracotomy was most frequently performed (*n* = 17, 32%), followed by laparotomy (*n* = 12, 23%), fasciotomy (*n* = 9, 17%), and surgical revision of ECMO (*n* = 8, 15%) (Figure 2).

**Table 2:** Baseline, ECMO, and surgical characteristics of surgical patients.

	**Total**	**Mortality**	**No mortality**	***P*-value**
	**(*N* = 32, 14%)**	**(*N* = 16, 50%)**	**(*N* = 16, 50%)**	
Male	18 (56%)	6 (38)	12 (75)	0.033^*^
Gestational age at birth	38.79 (31.71–41.71)	38.57 (34.14–40.14)	38.71 (31.71–41.71)	0.482
Birthweight	2700 (1800–4134)	2670 (2075–3690)	2705 (1800–4134)	0.862
Weight at start of ECMO^†^	14 (2.55–75)	14.3 (2.55–60)	16.10 (2.84–75)	0.577
Age at start of ECMO categorical^†^				0.909
Premature <28 days	0	0	0	
At term <28 days	7 (22%)	4 (25%)	3 (19%)	
28 days−2years	4 (13%)	2 (12.5%)	2 (13%)	
>2 years	21 (66%)	10 (62.5%)	11 (69%)	
ECMO indication^†^				0.242
Neonatal-respiratory	6 (19%)	4 (25%)	2 (13%)	
Neonatal-cardiac	0	0	0	
Neonatal-ECPR^‡^	1 (3%)	0	1 (6%)	
Pediatric-respiratory	13 (40%)	8 (50%)	5 (31%)	
Pediatric-cardiac	3 (9%)	0	3 (19%)	
Pediatric-ECPR^‡^	9 (28%)	4 (25%)	5 (31%)	
ECMO type^†^				1
VVDL	8 (25%)	4 (25%)	4 (25%)	
VA	16 (50%)	8 (50%)	8 (50%)	
VV	2 (6%)	1 (6.3%)	1 (6%)	
Multiple	6 (19%)	3 (19%)	3 (19%)	
ECMO duration^†^				0.758
<7 days	12 (38%)	5 (31%)	7 (44%)	
7–20 days	13 (41%)	7 (44%)	6 (38%)	
>20 days	7 (22%)	4 (25%)	3 (19%)	
ECMO run(s)^†^				1
1	30 (94%)	15 (94%)	15 (94%)	
2	2 (6%)	1 (6%)	1 (6%)	
3	0	0	0	
4	0	0	0	
Number of surgical interventions per patient				0.524
1	15 (47%)	6 (38%)	9 (56%)	
2	13 (40%)	8 (50%)	5 (31%)	
3	4 (13%)	2 (13%)	2 (13%)	
Total	53	28	25	
Thoracic surgery	21 (40%)	14	7	0.241
Abdominal surgery	12 (23%)	7	5	0.465
Vascular surgery	20 (38%)	7	13	0.389
Day in ECMO-run 1st intervention	6 (1–38)	6 (1–18)	6 (1–38)	0.422
Day in ECMO-run 2nd intervention	10 (2–42)	8 (2–19)	12 (2–42)	0.567
Day in ECMO-run 3rd intervention	19 (9–41)	17 (14–19)	21 (9–41)	0.653
Reason of surgery				0.061
Therapeutic	46 (87%)	22 (79%)	24 (96%)	
Diagnostic	7 (13%)	6 (21%)	1 (4%)	
Elective/emergency surgery				0.361
Elective	8 (15%)	6 (21%)	3 (12%)	
Emergency	45 (85%)	22 (79%)	22 (88%)	

*Data are reported as n (%) and median (minimum-maximum)*.

* *Indicates significance (p < 0.05)*.

† *Extracorporeal membrane oxygenation*.

‡ *Extracorporeal cardiopulmonary resuscitation*.

Six out of the 17 thoracotomies were performed to obtain a lung biopsy, five for intra-thoracic hemorrhage, two for pleural effusion and two for empyema. One emergency thoracotomy was performed for a tension pneumothorax, and one for secondary closure of the thorax following a tracheal reconstruction.

Six out of the 12 laparotomies were indicated for suspicion of an abdominal compartment syndrome. In one case, a second-look laparotomy was performed 2 days later following elevated lactic acid; this revealed extensive bowel ischemia. One patient required two laparotomy procedures on the same day because of hemorrhage 9 days after CDH correction. In this case ECMO was not started until 4 days after the CDH repair had taken place, following the development of fulminant sepsis. One laparotomy was performed for an anastomotic leakage of the bowel. The remaining two cases concerned acute laparotomy. In one case an abdominal compartment syndrome was suspected, in the other a volvulus was discovered.

Comparing survivors with non-survivors, it appeared that neither the categorical type of surgery nor the number of surgical procedures per patient was significantly different (Table 2).

#### Indications and Planning

Most surgical interventions were therapeutic (87%), as opposed to 13% being diagnostic (Table 2). One diagnostic laparotomy was performed following an ECPR ECMO procedure, and this revealed a volvulus. This patient did not survive. The other six diagnostic surgical procedures were lung biopsies; three were indicated to identify the cause of pneumonia, the other three intended to investigate the cause of pulmonary hypertension. In one case, the result of the lung biopsy led to immediate cessation of therapy because of alveolar capillary dysplasia. Four out of the other five patients who underwent a lung biopsy procedure did not survive (Table 2). Death was in these cases not directly related to the surgical procedure. Comparison of the mortality rate related to either diagnostic or therapeutic surgical procedures did not show a significant difference. The majority of surgical procedures took place in an emergency setting (85%), as opposed to 15% being elective. Six of these procedures were the abovementioned diagnostic lung biopsies. In one case a tracheal reconstruction took place on ECMO due to an obstructive trachea and in one case an arterial line was placed by surgical cut down. The mortality rate was not significantly different between elective and emergency surgical cases (Table 2).

#### Outcome

Complications after surgery, including mortality, were seen in 88% of cases. The total in-hospital mortality was 50% in the surgical group. One quarter of deaths occurred after cessation of ECMO. Futility was the most reported reason of death (22%), resulting in cessation of therapy after multidisciplinary consensus was reached. Pulmonary failure was noted as reason of death in 13% of cases, and cardiovascular failure accounted for 9% of cases. Additional information concerning cause of death in the surgical cases can be found in the Supplementary Material.

## Discussion

To our knowledge, this is the first study describing outcomes of neonatal and pediatric patients undergoing a surgical intervention while on ECMO, apart from CDH repair, and cardiac surgery.

We found that 14% of the patients who received ECMO-treatment in the study period had undergone a surgical intervention while on ECMO, of whom more than half required multiple interventions. Thoracotomy was the most frequent intervention, followed by laparotomy, fasciotomy and surgical revision of ECMO cannulas. Complications occurred in 88% of surgical patients, associated with death in half of the cases. Other frequent complications included compartment syndrome and hemorrhage. The most common reason of death was futility, resulting in cessation of therapy. Patients who underwent surgery on ECMO had a significantly longer ICU stay and total hospital stay compared to non-surgical patients.

These results suggest that patients who undergo surgery while on ECMO have a higher risk of complications compared to those not operated on. In our cohort, this risk did not lead to a significantly higher in-hospital mortality in surgical patients (50 vs. 41% *p* = 0.327). This may be related to the relatively small sample sizes, as is also seen in literature in adults (5). In the present study, one quarter of deaths in surgical patients occurred after cessation of ECMO-treatment, which demonstrates that the critical period of patients in need of surgery while on ECMO does not end with decannulation.

The proportion of neonates and children on ECMO in our cohort receiving surgery (14%) is relatively low in comparison with studies in adults, reporting incidences from 14 to 48% (5, 11). We hypothesize that this relatively low proportion is related to the relatively large group of neonates in our cohort who need ECMO for respiratory support directly post-partum, few of whom require surgical intervention during ECMO.

When comparing the ECMO-indication in surgical and non-surgical patients, a larger proportion within the non-surgical group is represented by neonates who are in need of ECMO due to respiratory failure (32% non-surgical vs. 19% surgical, *p* = 0.144). This patient group is known to have a relatively favorable prognosis (10). However, in our cohort we did not find a significant difference in mortality between the surgical and non-surgical group (50 vs. 41%, *p* = 0.327). We hypothesize that this is due to the fact that the neonatal-respiratory group represents a minority within our cohort (in total 68 out of 221 patients), and therefore does not significantly influence the mortality of either of the groups.

Various studies have focused on specific surgical interventions on ECMO such as cardiac catheterization, CDH correction and lung biopsies (13, 20, 21). We, however, investigated the surgical interventions in children on ECMO in general, and found a high complication rate as well as a high mortality rate in this group of patients. These findings can help improve the quality of counseling.

We searched the ELSO-registry but could not identify data on surgical interventions during ECMO. Though, complications including surgical site hemorrhage, cannulation site hemorrhage, and compartment syndrome were reported, numbers of surgical procedures appear not to be documented systematically (10). As we found that in our center approximately one in seven neonatal and pediatric patients on ECMO undergoes surgery—associated with a distinctly high complication rate—we advocate for the systematic registration of surgical interventions during ECMO in the future. Centralized registration will possibly lead to new insights and thereby influence clinical practice.

The retrospective nature of this study accounts for the possibility of missing data. The quality of data was especially poor concerning coagulation complications and transfusions in the perioperative period. Due to the large portion of missing data, analysis of these parameters was not possible. Colleagues Erdem et al. described coagulation complications on ECMO, however the relationship of these complications to surgical interventions during ECMO was not further analyzed in this study (22).

Follow-up was limited to either death or first hospital discharge after ECMO-treatment; we did not inventory whether complications had occurred thereafter. IJsselstijn et al. already stressed the importance of multidisciplinary long-term follow-up with a standardized approach, which is now being implemented in our center (23). Even though our cohort stems from the largest neonatal and pediatric ECMO-center in the Netherlands, we found it to be too small and heterogeneous for a clinically significant prediction model. In order to achieve a cohort fit for a prediction model, a multi-center study design will be necessary in order to include a sufficient number of patients. Large multi-center studies have reported high rates of complications due to either bleeding or thrombosis, warranting future studies exploring new coagulation strategies during ECMO (24, 25). Likewise, future multi-center studies focusing on surgical procedures during ECMO could lead to identification of possible predictive variables for surgical outcome. ECMO-treatment is an evolving field of medical practice in which new instruments and techniques are frequently introduced. Consequently, it is plausible that during the 10 years of inclusion in the present study ECMO techniques, practice and the supportive care have changed, although, recent results suggest that the transition from roller to centrifugal pump techniques has not significantly influenced outcome (22).

In conclusion, approximately one in seven neonatal and pediatric patients required a surgical intervention during ECMO-treatment, 88% of them developed complications. In-hospital mortality after surgery on ECMO was 50%, which was not significantly higher than in non-surgical patients. These results should be taken into account in counseling.

## Data Availability Statement

The original contributions presented in the study are included in the article/Supplementary Material, further inquiries can be directed to the corresponding author/s.

## Ethics Statement

The studies involving human participants were reviewed and approved by Daily Board of the Medical Ethics Committee Erasmus MC. Written informed consent from the participants' legal guardian/next of kin was not required to participate in this study in accordance with the national legislation and the institutional requirements.

## Author Contributions

JS and RH contributed to conception and design of the study. CK organized the database, performed case study, and wrote the first draft of the manuscript. CK and SH performed data analysis. CK, SH, JS, and RH drafted the manuscript for important intellectual content. JS, RW, and DT contributed to revising the manuscript and gave final approval. All authors contributed to the article and approved the submitted version.

## Conflict of Interest

The authors declare that the research was conducted in the absence of any commercial or financial relationships that could be construed as a potential conflict of interest.
