# Supplementary material for: Surgery in Neonatal and Pediatric ECMO Patients Other Than Congenital Diaphragmatic Hernia Repair: A 10-Year Experience
Source: Front Pediatr. 2021 May 4;9:660647. doi: 10.3389/fped.2021.660647 (PMC8129514; doi:10.3389/fped.2021.660647)
Supplement: Supplementary file 1 [file Table_1.docx]

| **Reason of death categorical** | **Cause of death** |
| --- | --- |
| futility | Pulmonary interstitial glycogenosis |
| futility | Persistent circulatory failure following ECPR, infaust prognosis |
| futility | Extensive bowel necrosis in large cell anaplastic lymphoma |
| futility | Irreversible neurological deterioration after ECPR for a volvulus |
| futility | ARDS in patient with beta-thalassemia |
| futility | Alveolar Capillary Dysplasia |
| futility | Severe pulmonary hypertension without diagnosis |
| futility | Cerebral herniation in Addisonian crisis |
| pulmonary | Bilateral pneumonia with necrosis |
| pulmonary | H1N1 pneumonia with fungus |
| pulmonary | Pulmonary hypertension with recurrent pneumonia in patient with Turner mosaic |
| cardiovascular | Persistent circulatory failure after ECPR after near-drowning |
| cardiovascular | Persistent circulatory failure after ECPR after near-drowning |
| cardiovascular | Irreversible arrhythmia in patient with non-Hodgkin lymphoma |
| neurological | Traumatic brain injury |
| neurological | Recurrent intra-cerebral hemorrhage |
